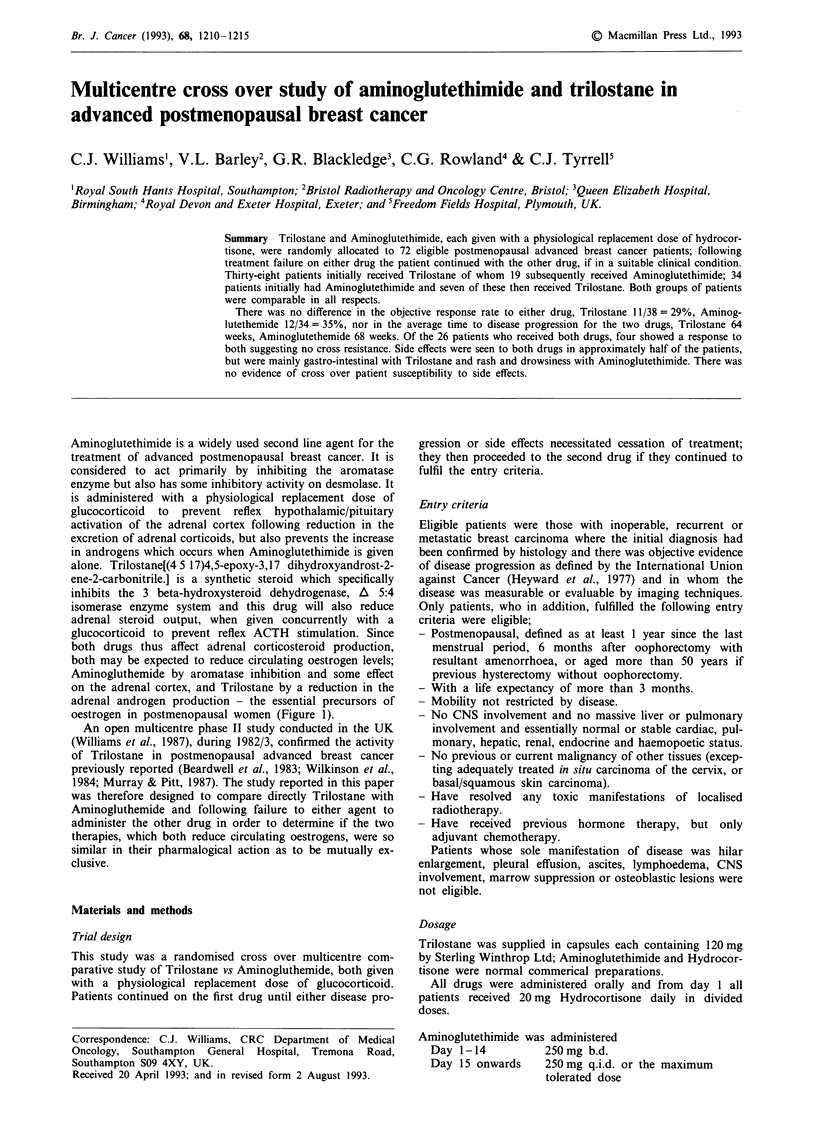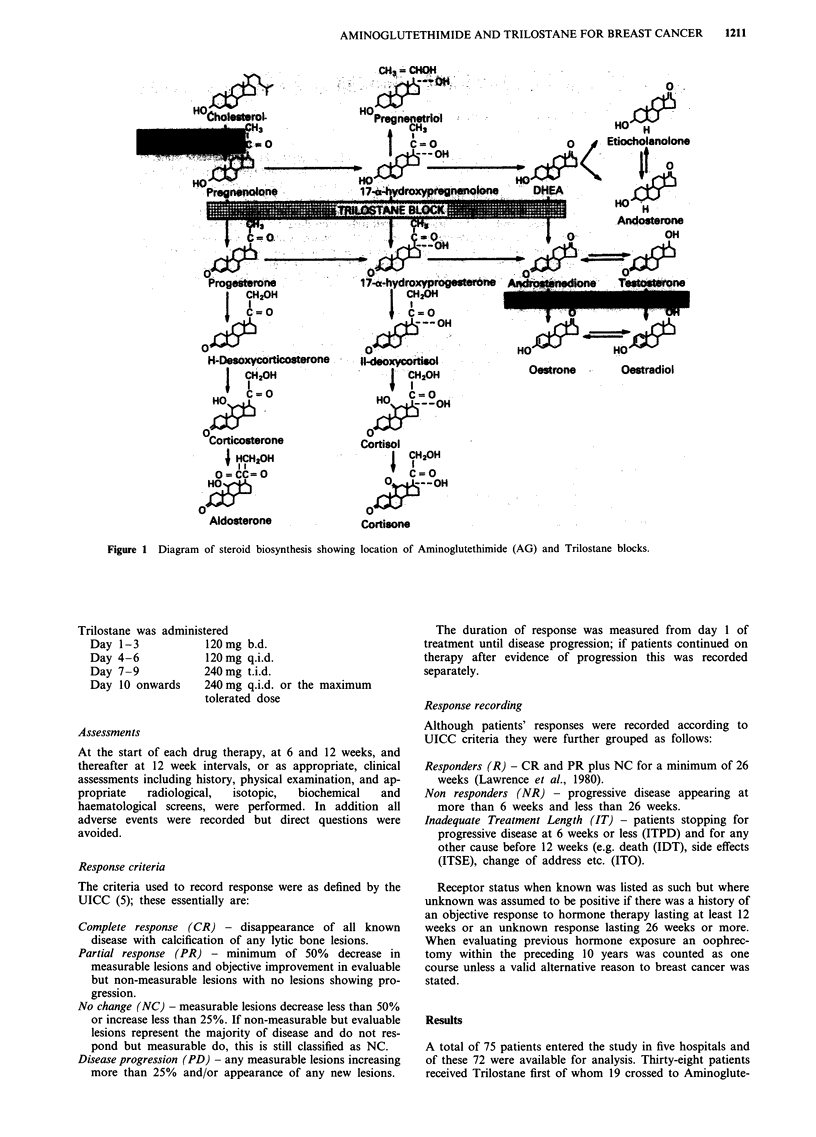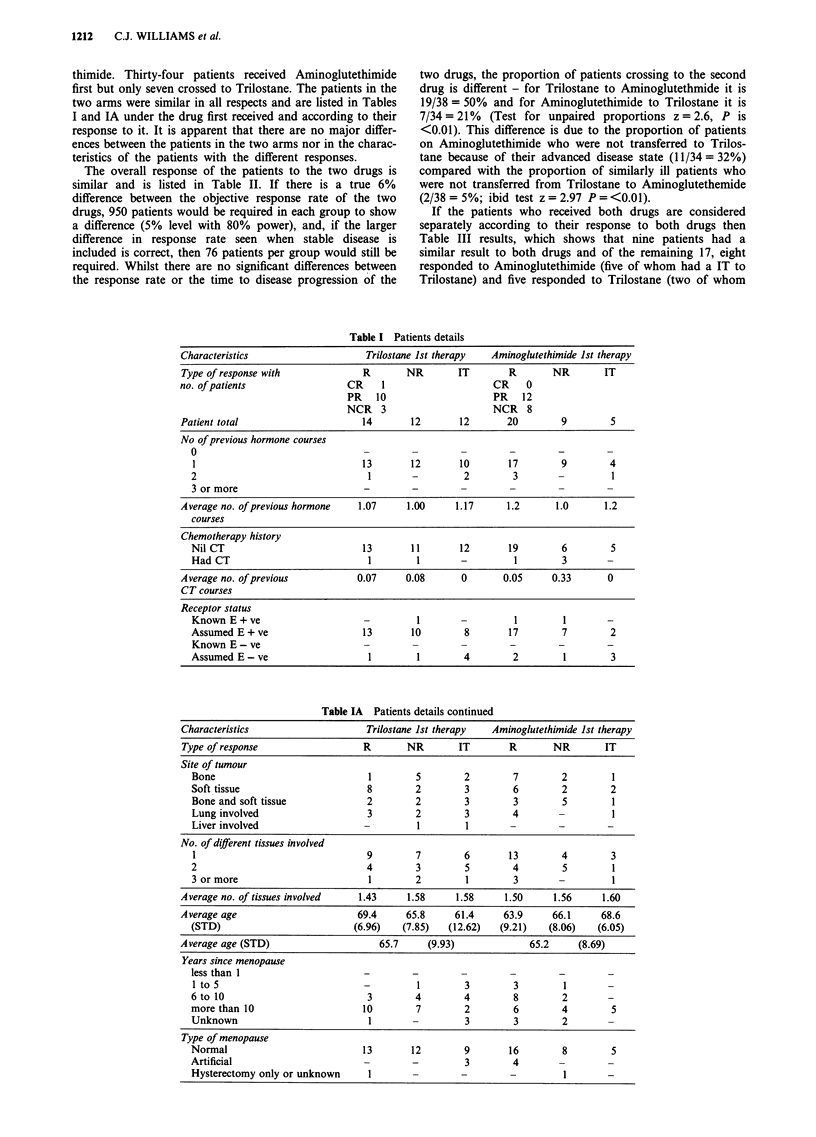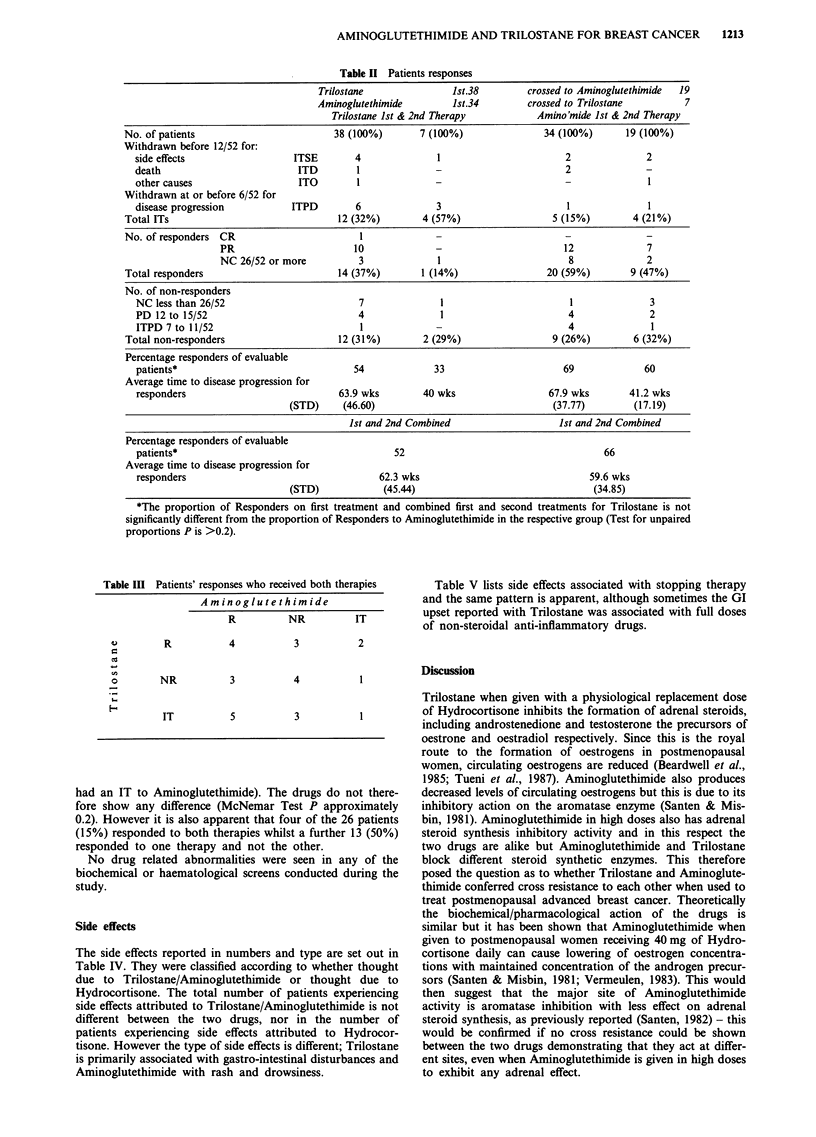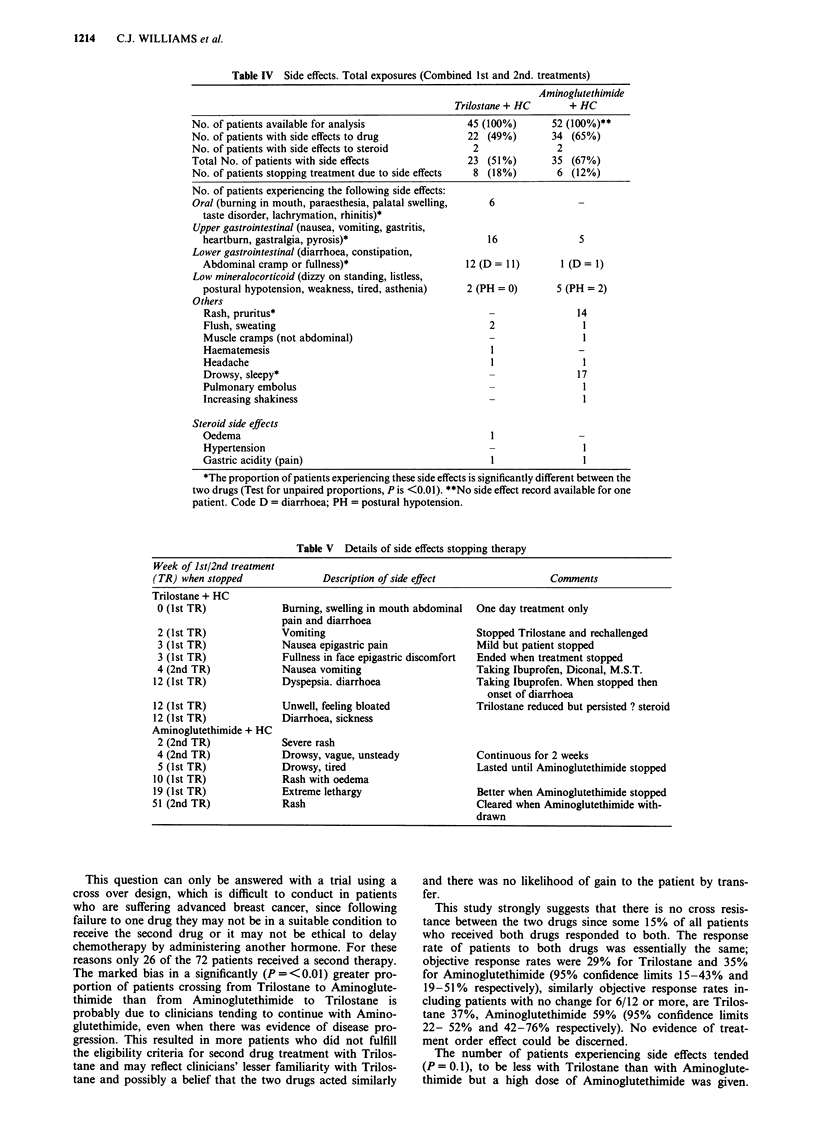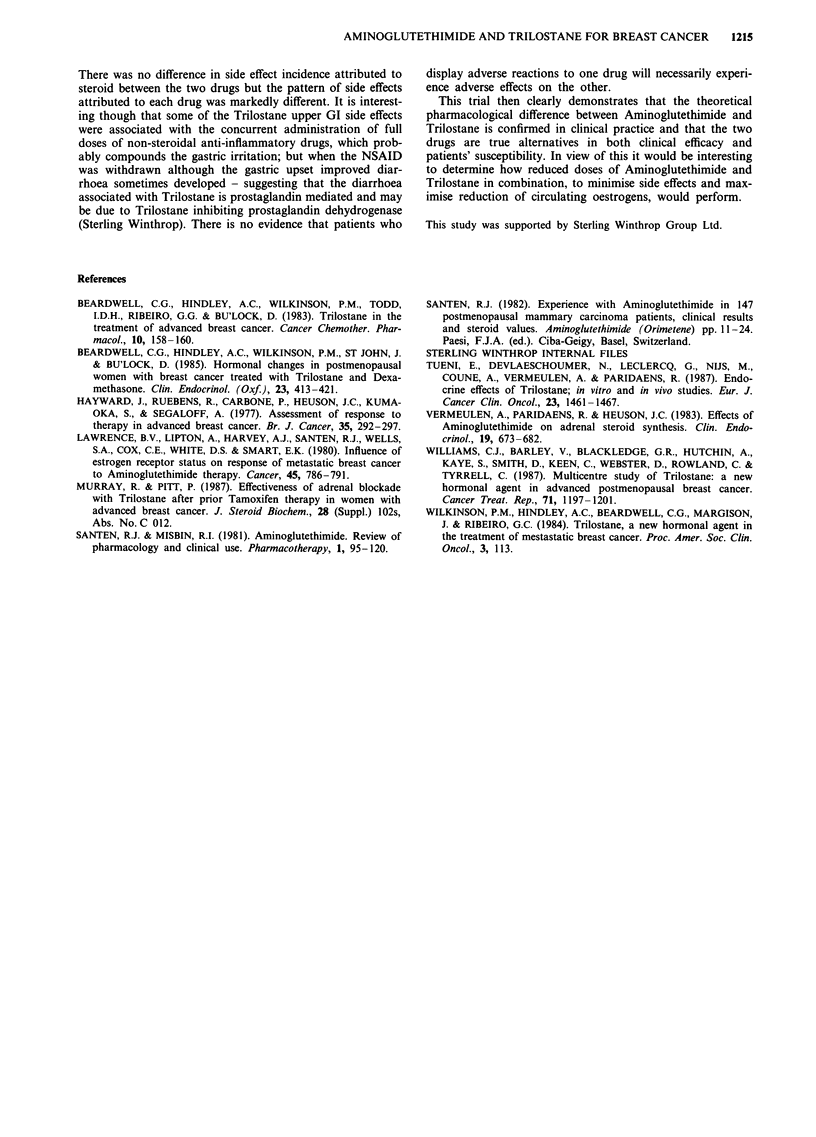# Multicentre cross over study of aminoglutethimide and trilostane in advanced postmenopausal breast cancer.

**DOI:** 10.1038/bjc.1993.506

**Published:** 1993-12

**Authors:** C. J. Williams, V. L. Barley, G. R. Blackledge, C. G. Rowland, C. J. Tyrrell

**Affiliations:** Royal South Hants Hospital, Southampton, UK.

## Abstract

Trilostane and Aminoglutethimide, each given with a physiological replacement dose of hydrocortisone, were randomly allocated to 72 eligible postmenopausal advanced breast cancer patients; following treatment failure on either drug the patient continued with the other drug, if in a suitable clinical condition. Thirty-eight patients initially received Trilostane of whom 19 subsequently received Aminoglutethimide; 34 patients initially had Aminoglutethimide and seven of these then received Trilostane. Both groups of patients were comparable in all respects. There was no difference in the objective response rate to either drug, Trilostane 11/38 = 29%, Aminoglutethimide 12/34 = 35%, nor in the average time to disease progression for the two drugs, Trilostane 64 weeks, Aminoglutethimide 68 weeks. Of the 26 patients who received both drugs, four showed a response to both suggesting no cross resistance. Side effects were seen to both drugs in approximately half of the patients, but were mainly gastro-intestinal with Trilostane and rash and drowsiness with Aminoglutethimide. There was no evidence of cross over patient susceptibility to side effects.


Br. J. Cancer (1993), 68, 1210–1215 © Macmillan Press Ltd., 1993

### Multicentre cross over study of aminoglutethimide and trilostane in advanced postmenopausal breast cancer

C.J. Williams[1], V.L. Barley[2], G.R. Blackledge[3], C.G. Rowland[4] & C.J. Tyrrell[5]

[1]*Royal South Hants Hospital, Southampton;* [2]*Bristol Radiotherapy and Oncology Centre, Bristol;* [3]*Queen Elizabeth Hospital, Birmingham;* [4]*Royal Devon and Exeter Hospital, Exeter; and* [5]*Freedom Fields Hospital, Plymouth, UK.*

**Summary** Trilostane and Aminoglutethimide, each given with a physiological replacement dose of hydrocortisone, were randomly allocated to 72 eligible postmenopausal advanced breast cancer patients; following treatment failure on either drug the patient continued with the other drug, if in a suitable clinical condition. Thirty-eight patients initially received Trilostane of whom 19 subsequently received Aminoglutethimide; 34 patients initially had Aminoglutethimide and seven of these then received Trilostane. Both groups of patients were comparable in all respects.

There was no difference in the objective response rate to either drug, Trilostane 11/38 = 29%, Aminoglutethemide 12/34 = 35%, nor in the average time to disease progression for the two drugs, Trilostane 64 weeks, Aminoglutethemide 68 weeks. Of the 26 patients who received both drugs, four showed a response to both suggesting no cross resistance. Side effects were seen to both drugs in approximately half of the patients, but were mainly gastro-intestinal with Trilostane and rash and drowsiness with Aminoglutethimide. There was no evidence of cross over patient susceptibility to side effects.

Aminoglutethimide is a widely used second line agent for the treatment of advanced postmenopausal breast cancer. It is considered to act primarily by inhibiting the aromatase enzyme but also has some inhibitory activity on desmolase. It is administered with a physiological replacement dose of glucocorticoid to prevent reflex hypothalamic/pituitary activation of the adrenal cortex following reduction in the excretion of adrenal corticoids, but also prevents the increase in androgens which occurs when Aminoglutethimide is given alone. Trilostane[(4 5 17)4,5-epoxy-3,17 dihydroxyandrost-2-ene-2-carbonitrile.] is a synthetic steroid which specifically inhibits the 3 beta-hydroxysteroid dehydrogenase, Δ 5:4 isomerase enzyme system and this drug will also reduce adrenal steroid output, when given concurrently with a glucocorticoid to prevent reflex ACTH stimulation. Since both drugs thus affect adrenal corticosteroid production, both may be expected to reduce circulating oestrogen levels; Aminogluthemide by aromatase inhibition and some effect on the adrenal cortex, and Trilostane by a reduction in the adrenal androgen production – the essential precursors of oestrogen in postmenopausal women (Figure 1).

An open multicentre phase II study conducted in the UK (Williams *et al.*, 1987), during 1982/3, confirmed the activity of Trilostane in postmenopausal advanced breast cancer previously reported (Beardwell *et al.*, 1983; Wilkinson *et al.*, 1984; Murray & Pitt, 1987). The study reported in this paper was therefore designed to compare directly Trilostane with Aminogluthemide and following failure to either agent to administer the other drug in order to determine if the two therapies, which both reduce circulating oestrogens, were so similar in their pharmalogical action as to be mutually exclusive.

### Materials and methods

### *Trial design*

This study was a randomised cross over multicentre comparative study of Trilostane *vs* Aminogluthemide, both given with a physiological replacement dose of glucocorticoid. Patients continued on the first drug until either disease progression or side effects necessitated cessation of treatment; they then proceeded to the second drug if they continued to fulfil the entry criteria.

### *Entry criteria*

Eligible patients were those with inoperable, recurrent or metastatic breast carcinoma where the initial diagnosis had been confirmed by histology and there was objective evidence of disease progression as defined by the International Union against Cancer (Heyward *et al.*, 1977) and in whom the disease was measurable or evaluable by imaging techniques. Only patients, who in addition, fulfilled the following entry criteria were eligible;

- Postmenopausal, defined as at least 1 year since the last menstrual period, 6 months after oophorectomy with resultant amenorrhoea, or aged more than 50 years if previous hysterectomy without oophorectomy.
- With a life expectancy of more than 3 months.
- Mobility not restricted by disease.
- No CNS involvement and no massive liver or pulmonary involvement and essentially normal or stable cardiac, pulmonary, hepatic, renal, endocrine and haemopoetic status.
- No previous or current malignancy of other tissues (excepting adequately treated *in situ* carcinoma of the cervix, or basal/squamous skin carcinoma).
- Have resolved any toxic manifestations of localised radiotherapy.
- Have received previous hormone therapy, but only adjuvant chemotherapy.

Patients whose sole manifestation of disease was hilar enlargement, pleural effusion, ascites, lymphoedema, CNS involvement, marrow suppression or osteoblastic lesions were not eligible.

### *Dosage*

Trilostane was supplied in capsules each containing 120 mg by Sterling Winthrop Ltd; Aminoglutethimide and Hydrocortisone were normal commerical preparations.

All drugs were administered orally and from day 1 all patients received 20 mg Hydrocortisone daily in divided doses.

Aminoglutethimide was administered

| | |
|---|---|
| Day 1–14 | 250 mg b.d. |
| Day 15 onwards | 250 mg q.i.d. or the maximum tolerated dose |

Correspondence: C.J. Williams, CRC Department of Medical Oncology, Southampton General Hospital, Tremona Road, Southampton S09 4XY, UK.
Received 20 April 1993; and in revised form 2 August 1993.

**Figure 1** Diagram of steroid biosynthesis showing location of Aminoglutethimide (AG) and Trilostane blocks.

Trilostane was administered

| | |
|---|---|
| Day 1–3 | 120 mg b.d. |
| Day 4–6 | 120 mg q.i.d. |
| Day 7–9 | 240 mg t.i.d. |
| Day 10 onwards | 240 mg q.i.d. or the maximum tolerated dose |

### *Assessments*

At the start of each drug therapy, at 6 and 12 weeks, and thereafter at 12 week intervals, or as appropriate, clinical assessments including history, physical examination, and appropriate radiological, isotopic, biochemical and haematological screens, were performed. In addition all adverse events were recorded but direct questions were avoided.

### *Response criteria*

The criteria used to record response were as defined by the UICC (5); these essentially are:

*Complete response (CR)* – disappearance of all known disease with calcification of any lytic bone lesions.

*Partial response (PR)* – minimum of 50% decrease in measurable lesions and objective improvement in evaluable but non-measurable lesions with no lesions showing progression.

*No change (NC)* – measurable lesions decrease less than 50% or increase less than 25%. If non-measurable but evaluable lesions represent the majority of disease and do not respond but measurable do, this is still classified as NC.

*Disease progression (PD)* – any measurable lesions increasing more than 25% and/or appearance of any new lesions.

The duration of response was measured from day 1 of treatment until disease progression; if patients continued on therapy after evidence of progression this was recorded separately.

### *Response recording*

Although patients' responses were recorded according to UICC criteria they were further grouped as follows:

*Responders (R)* – CR and PR plus NC for a minimum of 26 weeks (Lawrence *et al.*, 1980).

*Non responders (NR)* – progressive disease appearing at more than 6 weeks and less than 26 weeks.

*Inadequate Treatment Length (IT)* – patients stopping for progressive disease at 6 weeks or less (ITPD) and for any other cause before 12 weeks (e.g. death (IDT), side effects (ITSE), change of address etc. (ITO).

Receptor status when known was listed as such but where unknown was assumed to be positive if there was a history of an objective response to hormone therapy lasting at least 12 weeks or an unknown response lasting 26 weeks or more. When evaluating previous hormone exposure an oophrectomy within the preceding 10 years was counted as one course unless a valid alternative reason to breast cancer was stated.

### Results

A total of 75 patients entered the study in five hospitals and of these 72 were available for analysis. Thirty-eight patients received Trilostane first of whom 19 crossed to Aminoglutethimide. Thirty-four patients received Aminoglutethimide first but only seven crossed to Trilostane. The patients in the two arms were similar in all respects and are listed in Tables I and IA under the drug first received and according to their response to it. It is apparent that there are no major differences between the patients in the two arms nor in the characteristics of the patients with the different responses.

The overall response of the patients to the two drugs is similar and is listed in Table II. If there is a true 6% difference between the objective response rate of the two drugs, 950 patients would be required in each group to show a difference (5% level with 80% power), and, if the larger difference in response rate seen when stable disease is included is correct, then 76 patients per group would still be required. Whilst there are no significant differences between the response rate or the time to disease progression of the two drugs, the proportion of patients crossing to the second drug is different – for Trilostane to Aminoglutethmide it is 19/38 = 50% and for Aminoglutethimide to Trilostane it is 7/34 = 21% (Test for unpaired proportions $z = 2.6$, $P$ is $<0.01$). This difference is due to the proportion of patients on Aminoglutethimide who were not transferred to Trilostane because of their advanced disease state (11/34 = 32%) compared with the proportion of similarly ill patients who were not transferred from Trilostane to Aminoglutethemide (2/38 = 5%; ibid test $z = 2.97$ $P = <0.01$).

If the patients who received both drugs are considered separately according to their response to both drugs then Table III results, which shows that nine patients had a similar result to both drugs and of the remaining 17, eight responded to Aminoglutethimide (five of whom had a IT to Trilostane) and five responded to Trilostane (two of whom

**Table I** Patients details

| *Characteristics* | *Trilostane 1st therapy* | | | *Aminoglutethimide 1st therapy* | | |
|---|---|---|---|---|---|---|
| *Type of response with* | R | NR | IT | R | NR | IT |
| *no. of patients* | CR 1 | | | CR 0 | | |
| | PR 10 | | | PR 12 | | |
| | NCR 3 | | | NCR 8 | | |
| *Patient total* | 14 | 12 | 12 | 20 | 9 | 5 |
| *No of previous hormone courses* | | | | | | |
| 0 | – | – | – | – | – | – |
| 1 | 13 | 12 | 10 | 17 | 9 | 4 |
| 2 | 1 | – | 2 | 3 | – | 1 |
| 3 or more | – | – | – | – | – | – |
| *Average no. of previous hormone courses* | 1.07 | 1.00 | 1.17 | 1.2 | 1.0 | 1.2 |
| *Chemotherapy history* | | | | | | |
| Nil CT | 13 | 11 | 12 | 19 | 6 | 5 |
| Had CT | 1 | 1 | – | 1 | 3 | – |
| *Average no. of previous CT courses* | 0.07 | 0.08 | 0 | 0.05 | 0.33 | 0 |
| *Receptor status* | | | | | | |
| Known E + ve | – | 1 | – | 1 | 1 | – |
| Assumed E + ve | 13 | 10 | 8 | 17 | 7 | 2 |
| Known E – ve | – | – | – | – | – | – |
| Assumed E – ve | 1 | 1 | 4 | 2 | 1 | 3 |

**Table IA** Patients details continued

| *Characteristics* | *Trilostane 1st therapy* | | | *Aminoglutethimide 1st therapy* | | |
|---|---|---|---|---|---|---|
| *Type of response* | R | NR | IT | R | NR | IT |
| *Site of tumour* | | | | | | |
| Bone | 1 | 5 | 2 | 7 | 2 | 1 |
| Soft tissue | 8 | 2 | 3 | 6 | 2 | 2 |
| Bone and soft tissue | 2 | 2 | 3 | 3 | 5 | 1 |
| Lung involved | 3 | 2 | 3 | 4 | – | 1 |
| Liver involved | – | 1 | 1 | – | – | – |
| *No. of different tissues involved* | | | | | | |
| 1 | 9 | 7 | 6 | 13 | 4 | 3 |
| 2 | 4 | 3 | 5 | 4 | 5 | 1 |
| 3 or more | 1 | 2 | 1 | 3 | – | 1 |
| *Average no. of tissues involved* | 1.43 | 1.58 | 1.58 | 1.50 | 1.56 | 1.60 |
| *Average age* (STD) | 69.4 (6.96) | 65.8 (7.85) | 61.4 (12.62) | 63.9 (9.21) | 66.1 (8.06) | 68.6 (6.05) |
| *Average age* (STD) | 65.7 (9.93) | | | 65.2 (8.69) | | |
| *Years since menopause* | | | | | | |
| less than 1 | – | – | – | – | – | – |
| 1 to 5 | – | 1 | 3 | 3 | 1 | – |
| 6 to 10 | 3 | 4 | 4 | 8 | 2 | – |
| more than 10 | 10 | 7 | 2 | 6 | 4 | 5 |
| Unknown | 1 | – | 3 | 3 | 2 | – |
| *Type of menopause* | | | | | | |
| Normal | 13 | 12 | 9 | 16 | 8 | 5 |
| Artificial | – | – | 3 | 4 | – | – |
| Hysterectomy only or unknown | 1 | – | – | – | 1 | – |

**Table II** Patients responses

| | | Trilostane 1st.38<br>Aminoglutethimide 1st.34 | | crossed to Aminoglutethimide 19<br>crossed to Trilostane 7 | |
|---|---|---|---|---|---|
| | | *Trilostane 1st & 2nd Therapy* | | *Amino'mide 1st & 2nd Therapy* | |
| No. of patients | | 38 (100%) | 7 (100%) | 34 (100%) | 19 (100%) |
| Withdrawn before 12/52 for: | | | | | |
| side effects | ITSE | 4 | 1 | 2 | 2 |
| death | ITD | 1 | – | 2 | – |
| other causes | ITO | 1 | – | – | 1 |
| Withdrawn at or before 6/52 for disease progression | ITPD | 6 | 3 | 1 | 1 |
| Total ITs | | 12 (32%) | 4 (57%) | 5 (15%) | 4 (21%) |
| No. of responders | CR | 1 | – | – | – |
| | PR | 10 | – | 12 | 7 |
| | NC 26/52 or more | 3 | 1 | 8 | 2 |
| Total responders | | 14 (37%) | 1 (14%) | 20 (59%) | 9 (47%) |
| No. of non-responders | | | | | |
| NC less than 26/52 | | 7 | 1 | 1 | 3 |
| PD 12 to 15/52 | | 4 | 1 | 4 | 2 |
| ITPD 7 to 11/52 | | 1 | – | 4 | 1 |
| Total non-responders | | 12 (31%) | 2 (29%) | 9 (26%) | 6 (32%) |
| Percentage responders of evaluable patients* | | 54 | 33 | 69 | 60 |
| Average time to disease progression for responders | | 63.9 wks | 40 wks | 67.9 wks | 41.2 wks |
| | (STD) | (46.60) | | (37.77) | (17.19) |
| | | *1st and 2nd Combined* | | *1st and 2nd Combined* | |
| Percentage responders of evaluable patients* | | 52 | | 66 | |
| Average time to disease progression for responders | | 62.3 wks | | 59.6 wks | |
| | (STD) | (45.44) | | (34.85) | |

*The proportion of Responders on first treatment and combined first and second treatments for Trilostane is not significantly different from the proportion of Responders to Aminoglutethimide in the respective group (Test for unpaired proportions $P$ is $>0.2$).

**Table III** Patients' responses who received both therapies

| | | Aminoglutethimide | | |
|---|---|---|---|---|
| | | R | NR | IT |
| Trilostane | R | 4 | 3 | 2 |
| | NR | 3 | 4 | 1 |
| | IT | 5 | 3 | 1 |

had an IT to Aminoglutethimide). The drugs do not therefore show any difference (McNemar Test $P$ approximately 0.2). However it is also apparent that four of the 26 patients (15%) responded to both therapies whilst a further 13 (50%) responded to one therapy and not the other.

No drug related abnormalities were seen in any of the biochemical or haematological screens conducted during the study.

### Side effects

The side effects reported in numbers and type are set out in Table IV. They were classified according to whether thought due to Trilostane/Aminoglutethimide or thought due to Hydrocortisone. The total number of patients experiencing side effects attributed to Trilostane/Aminoglutethimide is not different between the two drugs, nor in the number of patients experiencing side effects attributed to Hydrocortisone. However the type of side effects is different; Trilostane is primarily associated with gastro-intestinal disturbances and Aminoglutethimide with rash and drowsiness.

Table V lists side effects associated with stopping therapy and the same pattern is apparent, although sometimes the GI upset reported with Trilostane was associated with full doses of non-steroidal anti-inflammatory drugs.

### Discussion

Trilostane when given with a physiological replacement dose of Hydrocortisone inhibits the formation of adrenal steroids, including androstenedione and testosterone the precursors of oestrone and oestradiol respectively. Since this is the royal route to the formation of oestrogens in postmenopausal women, circulating oestrogens are reduced (Beardwell *et al.*, 1985; Tueni *et al.*, 1987). Aminoglutethimide also produces decreased levels of circulating oestrogens but this is due to its inhibitory action on the aromatase enzyme (Santen & Misbin, 1981). Aminoglutethimide in high doses also has adrenal steroid synthesis inhibitory activity and in this respect the two drugs are alike but Aminoglutethimide and Trilostane block different steroid synthetic enzymes. This therefore posed the question as to whether Trilostane and Aminoglutethimide conferred cross resistance to each other when used to treat postmenopausal advanced breast cancer. Theoretically the biochemical/pharmacological action of the drugs is similar but it has been shown that Aminoglutethimide when given to postmenopausal women receiving 40 mg of Hydrocortisone daily can cause lowering of oestrogen concentrations with maintained concentration of the androgen precursors (Santen & Misbin, 1981; Vermeulen, 1983). This would then suggest that the major site of Aminoglutethimide activity is aromatase inhibition with less effect on adrenal steroid synthesis, as previously reported (Santen, 1982) – this would be confirmed if no cross resistance could be shown between the two drugs demonstrating that they act at different sites, even when Aminoglutethimide is given in high doses to exhibit any adrenal effect.

**Table IV** Side effects. Total exposures (Combined 1st and 2nd. treatments)

| | *Trilostane + HC* | *Aminoglutethimide + HC* |
|---|---|---|
| No. of patients available for analysis | 45 (100%) | 52 (100%)** |
| No. of patients with side effects to drug | 22 (49%) | 34 (65%) |
| No. of patients with side effects to steroid | 2 | 2 |
| Total No. of patients with side effects | 23 (51%) | 35 (67%) |
| No. of patients stopping treatment due to side effects | 8 (18%) | 6 (12%) |
| No. of patients experiencing the following side effects: | | |
| *Oral* (burning in mouth, paraesthesia, palatal swelling, taste disorder, lachrymation, rhinitis)* | 6 | – |
| *Upper gastrointestinal* (nausea, vomiting, gastritis, heartburn, gastralgia, pyrosis)* | 16 | 5 |
| *Lower gastrointestinal* (diarrhoea, constipation, Abdominal cramp or fullness)* | 12 (D = 11) | 1 (D = 1) |
| *Low mineralocorticoid* (dizzy on standing, listless, postural hypotension, weakness, tired, asthenia) | 2 (PH = 0) | 5 (PH = 2) |
| *Others* | | |
| Rash, pruritus* | – | 14 |
| Flush, sweating | 2 | 1 |
| Muscle cramps (not abdominal) | – | 1 |
| Haematemesis | 1 | – |
| Headache | 1 | 1 |
| Drowsy, sleepy* | – | 17 |
| Pulmonary embolus | – | 1 |
| Increasing shakiness | – | 1 |
| *Steroid side effects* | | |
| Oedema | 1 | – |
| Hypertension | – | 1 |
| Gastric acidity (pain) | 1 | 1 |

*The proportion of patients experiencing these side effects is significantly different between the two drugs (Test for unpaired proportions, $P$ is $<0.01$). **No side effect record available for one patient. Code D = diarrhoea; PH = postural hypotension.

**Table V** Details of side effects stopping therapy

| *Week of 1st/2nd treatment (TR) when stopped* | *Description of side effect* | *Comments* |
|---|---|---|
| Trilostane + HC | | |
| 0 (1st TR) | Burning, swelling in mouth abdominal pain and diarrhoea | One day treatment only |
| 2 (1st TR) | Vomiting | Stopped Trilostane and rechallenged |
| 3 (1st TR) | Nausea epigastric pain | Mild but patient stopped |
| 3 (1st TR) | Fullness in face epigastric discomfort | Ended when treatment stopped |
| 4 (2nd TR) | Nausea vomiting | Taking Ibuprofen, Diconal, M.S.T. |
| 12 (1st TR) | Dyspepsia. diarrhoea | Taking Ibuprofen. When stopped then onset of diarrhoea |
| 12 (1st TR) | Unwell, feeling bloated | Trilostane reduced but persisted ? steroid |
| 12 (1st TR) | Diarrhoea, sickness | |
| Aminoglutethimide + HC | | |
| 2 (2nd TR) | Severe rash | |
| 4 (2nd TR) | Drowsy, vague, unsteady | Continuous for 2 weeks |
| 5 (1st TR) | Drowsy, tired | Lasted until Aminoglutethimide stopped |
| 10 (1st TR) | Rash with oedema | |
| 19 (1st TR) | Extreme lethargy | Better when Aminoglutethimide stopped |
| 51 (2nd TR) | Rash | Cleared when Aminoglutethimide withdrawn |

This question can only be answered with a trial using a cross over design, which is difficult to conduct in patients who are suffering advanced breast cancer, since following failure to one drug they may not be in a suitable condition to receive the second drug or it may not be ethical to delay chemotherapy by administering another hormone. For these reasons only 26 of the 72 patients received a second therapy. The marked bias in a significantly ($P = <0.01$) greater proportion of patients crossing from Trilostane to Aminoglutethimide than from Aminoglutethimide to Trilostane is probably due to clinicians tending to continue with Aminoglutethimide, even when there was evidence of disease progression. This resulted in more patients who did not fulfill the eligibility criteria for second drug treatment with Trilostane and may reflect clinicians' lesser familiarity with Trilostane and possibly a belief that the two drugs acted similarly and there was no likelihood of gain to the patient by transfer.

This study strongly suggests that there is no cross resistance between the two drugs since some 15% of all patients who received both drugs responded to both. The response rate of patients to both drugs was essentially the same; objective response rates were 29% for Trilostane and 35% for Aminoglutethimide (95% confidence limits 15–43% and 19–51% respectively), similarly objective response rates including patients with no change for 6/12 or more, are Trilostane 37%, Aminoglutethimide 59% (95% confidence limits 22- 52% and 42–76% respectively). No evidence of treatment order effect could be discerned.

The number of patients experiencing side effects tended ($P = 0.1$), to be less with Trilostane than with Aminoglutethimide but a high dose of Aminoglutethimide was given.

There was no difference in side effect incidence attributed to steroid between the two drugs but the pattern of side effects attributed to each drug was markedly different. It is interesting though that some of the Trilostane upper GI side effects were associated with the concurrent administration of full doses of non-steroidal anti-inflammatory drugs, which probably compounds the gastric irritation; but when the NSAID was withdrawn although the gastric upset improved diarrhoea sometimes developed – suggesting that the diarrhoea associated with Trilostane is prostaglandin mediated and may be due to Trilostane inhibiting prostaglandin dehydrogenase (Sterling Winthrop). There is no evidence that patients who display adverse reactions to one drug will necessarily experience adverse effects on the other.

This trial then clearly demonstrates that the theoretical pharmacological difference between Aminoglutethimide and Trilostane is confirmed in clinical practice and that the two drugs are true alternatives in both clinical efficacy and patients' susceptibility. In view of this it would be interesting to determine how reduced doses of Aminoglutethimide and Trilostane in combination, to minimise side effects and maximise reduction of circulating oestrogens, would perform.

This study was supported by Sterling Winthrop Group Ltd.

### References

BEARDWELL, C.G., HINDLEY, A.C., WILKINSON, P.M., TODD, I.D.H., RIBEIRO, G.G. & BU'LOCK, D. (1983). Trilostane in the treatment of advanced breast cancer. *Cancer Chemother. Pharmacol.*, **10**, 158–160.

BEARDWELL, C.G., HINDLEY, A.C., WILKINSON, P.M., ST JOHN, J. & BU'LOCK, D. (1985). Hormonal changes in postmenopausal women with breast cancer treated with Trilostane and Dexamethasone. *Clin. Endocrinol. (Oxf.)*, **23**, 413–421.

HAYWARD, J., RUEBENS, R., CARBONE, P., HEUSON, J.C., KUMAOKA, S., & SEGALOFF, A. (1977). Assessment of response to therapy in advanced breast cancer. *Br. J. Cancer*, **35**, 292–297.

LAWRENCE, B.V., LIPTON, A., HARVEY, A.J., SANTEN, R.J., WELLS, S.A., COX, C.E., WHITE, D.S. & SMART, E.K. (1980). Influence of estrogen receptor status on response of metastatic breast cancer to Aminoglutethimide therapy. *Cancer*, **45**, 786–791.

MURRAY, R. & PITT, P. (1987). Effectiveness of adrenal blockade with Trilostane after prior Tamoxifen therapy in women with advanced breast cancer. *J. Steroid Biochem.*, **28** (Suppl.) 102s, Abs. No. C 012.

SANTEN, R.J. & MISBIN, R.I. (1981). Aminoglutethimide. Review of pharmacology and clinical use. *Pharmacotherapy*, **1**, 95–120.

SANTEN, R.J. (1982). Experience with Aminoglutethimide in 147 postmenopausal mammary carcinoma patients, clinical results and steroid values. *Aminoglutethimide (Orimetene)* pp. 11–24. Paesi, F.J.A. (ed.). Ciba-Geigy, Basel, Switzerland.

STERLING WINTHROP INTERNAL FILES

TUENI, E., DEVLAESCHOUMER, N., LECLERCQ, G., NIJS, M., COUNE, A., VERMEULEN, A. & PARIDAENS, R. (1987). Endocrine effects of Trilostane; *in vitro* and *in vivo* studies. *Eur. J. Cancer Clin. Oncol.*, **23**, 1461–1467.

VERMEULEN, A., PARIDAENS, R. & HEUSON, J.C. (1983). Effects of Aminoglutethimide on adrenal steroid synthesis. *Clin. Endocrinol.*, **19**, 673–682.

WILLIAMS, C.J., BARLEY, V., BLACKLEDGE, G.R., HUTCHIN, A., KAYE, S., SMITH, D., KEEN, C., WEBSTER, D., ROWLAND, C. & TYRRELL, C. (1987). Multicentre study of Trilostane: a new hormonal agent in advanced postmenopausal breast cancer. *Cancer Treat. Rep.*, **71**, 1197–1201.

WILKINSON, P.M., HINDLEY, A.C., BEARDWELL, C.G., MARGISON, J. & RIBEIRO, G.C. (1984). Trilostane, a new hormonal agent in the treatment of mestastatic breast cancer. *Proc. Amer. Soc. Clin. Oncol.*, **3**, 113.